# Asian Dust Storm Elevates Children’s Respiratory Health Risks: A Spatiotemporal Analysis of Children’s Clinic Visits across Taipei (Taiwan)

**DOI:** 10.1371/journal.pone.0041317

**Published:** 2012-07-27

**Authors:** Hwa-Lung Yu, Lung-Chang Chien, Chiang-Hsing Yang

**Affiliations:** 1 Department of Bioenvironmental Systems Engineering, National Taiwan University, Taipei, Taiwan; 2 Department of Internal Medicine, Division of Health Behavior Research, Washington University School of Medicine, St. Louis, Missouri, United States of America; 3 Department of Health Care Management, National Taipei University of Nursing and Health Sciences, Taipei, Taiwan; Kenya Medical Research Institute - Wellcome Trust Research Programme, Kenya

## Abstract

Concerns have been raised about the adverse impact of Asian dust storms (ADS) on human health; however, few studies have examined the effect of these events on children’s health. Using databases from the Taiwan National Health Insurance and Taiwan Environmental Protection Agency, this study investigates the documented daily visits of children to respiratory clinics during and after ADS that occurred from 1997 to 2007 among 12 districts across Taipei City by applying a Bayesian structural additive regressive model controlled for spatial and temporal patterns. This study finds that the significantly impact of elevated children’s respiratory clinic visits happened after ADS. Five of the seven lagged days had increasing percentages of relative rate, which was consecutively elevated from a 2-day to a 5-day lag by 0.63%∼2.19% for preschool children (i.e., 0∼6 years of age) and 0.72%∼3.17% for school children (i.e., 7∼14 years of age). The spatial pattern of clinic visits indicated that geographical heterogeneity was possibly associated with the clinic’s location and accessibility. Moreover, day-of-week effects were elevated on Monday, Friday, and Saturday. We concluded that ADS may significantly increase the risks of respiratory diseases consecutively in the week after exposure, especially in school children.

## Introduction

Every winter and spring season, Asian dust storms (ADS) are frequently a major health problem in many cities throughout the world. Annual dust emissions total approximately 43 million tons, with springtime dust emissions accounting for almost half of the annual totals [1]. ADS usually originate in the deserts of Mongolia and China, lift soil particles into the atmosphere, and carry them east and southeast towards Korea, Japan, Taiwan, and even the Philippines [2], [3]. In addition to highly-elevated ambient particulate matter (PM) concentration, the PM composition during ADS differs from that during non-ADS periods. Reports from central Taiwan show that the concentration of the crustal elements and sea salt species in PM_2.5-10_ (i.e., particulate matters with granulometric diameter between 2.5 and 10 µm) during ADS exceeds the mean concentration of these same entities during non-ADS periods by at least 2 units of factors [4]. Moreover, during ADS in China, higher PM concentrations were measured in Korea and Japan, and the ADS particles were also observed to be rich in some elements that include aluminum, iron, and calcium [5]–[7].

Several environmental epidemiologic studies have demonstrated evidence of an adverse relationship between airborne particles and human health. Airborne particles uniquely impact the mortality and hospitalization rates of several diseases, most notably respiratory diseases. Most research has concluded that the effects of PM, especially composed of fine particles, are more severe than that of other air pollutants because they can be inhaled deeply into the lungs [8]. A European study revealed that each 10 µg/m^3^ increase of PM_10_ (i.e., particulate matters with granulometric diameter less than 10 µm) raises the relative rate of clinic visit by 1.20% for asthma and 1.00% for chronic obstructive pulmonary disease [9]. In addition, the effect of fine particulate air pollution on mortality may contribute to higher instances of stroke and respiratory deaths [10]. Besides PM, the adverse health impact of CO, O_3_, NO_2_ and SO_2_ has also been well-documented historically [11]–[13].

The health impact of ADS has been discussed by comparing mortality rates or emergency room admissions between ADS and non-ADS events [14], [15]. In addition, researchers have investigated health related lag effects associated with ADS, and they have discovered deferred influence on hospital admissions [16]–[18]. Yang et al. [19] found a statistically significant association between ADS events and primary intracerebral hemorrhagic stroke admissions 3 days after the dust storms. ADS also increased the risk of both asthma and cerebrovascular admissions from a 1-day lag to a 3-day lag [20], [21]. Among the research population, children are particularly sensitive to airborne exposure [22]. However, few studies have focused on children’s health from the perspective of ADS events [23], [24]. Most of the previous studies regarding the health impact analysis of ADS utilize relatively few health observations, and therefore, inferences from these studies may be limited and conservative [25]. Chien et al. [24] showed the elevated rate of children’s respiratory clinic usage during one week following ADS; however, no temporal lag effect structure of health impact was discussed.

Geographic heterogeneity has been a salient factor in ambient pollutant distributions [26], [27] as well as its associations with health outcomes [12], [28]. Nevertheless, few studies have assessed the spatial variation of an ADS’s impact on human health. In order to address this issue, this study applies a unidirectional approach [29] under a spatiotemporal model framework to diagnose the space-time disparity of children’s respiratory clinic visits. The influence of ADS on children’s health was examined by considering temporal lag effects starting from the end of each ADS event as well as the spatial variation over study areas. This study specifically investigates the daily clinic visits of children with respiratory diseases in 12 districts in Taipei City from 1997 to 2007.

## Materials and Methods

### Children’s Clinic Data

Initiated in March 1995, Taiwan’s National Health Insurance (NHI) program contacted more than 97% of hospitals and clinics nationwide within its first year of inception, enrolling more than 96% of Taiwanese residents. The Taiwan National Health Research Institute maintains the NHI program database, and has established a standard procedure that assures the quality and accuracy of claims data [30]. The NHI database includes ambulatory care expenditures by visit as well as the registries of contracted medical facilities nationwide. The procedure and diagnostic codes are used to retrieve cause-specific data according to diagnosis-related groups or International Classification of Diseases, Ninth Revision, Clinical Modification (ICD-9-CM) classification codes by the Bureau of National Health Insurance. Regarding personal privacy and confidentiality, all individually identifiable health information has been encrypted prior to release, e.g., personal identification or hospital identification numbers.

For this study, the following respiratory diseases were highlighted: acute respiratory infections (ICD-9∶460–466, allergic rhinitis (ICD-9∶477), other diseases of upper respiratory tract (ICD-9∶478), pneumonia and influenza (ICD-9∶480–488), asthma (ICD-9∶493), bronchiectasis (ICD-9∶494), and extrinsic allergic alveolitis (ICD-9∶495). This study obtained a population-based database containing space-time data for clinic and hospital visits (i.e., hospital location and appointment times) for all-cause respiratory diseases of children under 14 years old in Taipei City from 1997–2007, including both ambulatory and emergency visits. We split these clinic visits data into preschool children (0∼6 years of age) and school children (7∼14 years of age) in this analysis.

### Dust Storm Data

**Figure 1:**
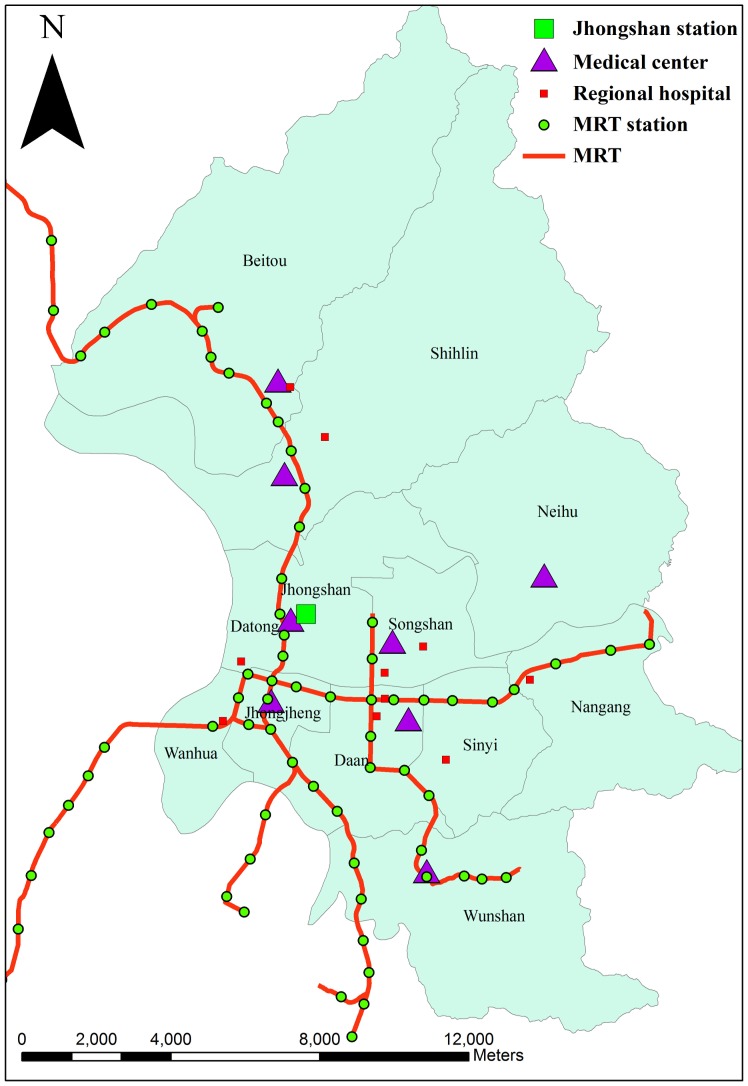
Spatial distribution of the districts, the medical centers, the MRT lines, and the Jhongshan air quality monitoring station in Taipei City.

ADS often occur in northern and northwestern China, impacting Taiwan only under certain atmospheric circumstances. Before 2000, the Department of Atmospheric Science at Chinese Culture University (CCU) was tasked with the responsibility of characterizing, defining, and monitoring ADS events in Taiwan. They came up with the following criteria for identifying an ADS event: 1) dust storm events with PM_10_ concentrations >100 µg/m^3^ observed by any air quality monitoring stations located in Wanli, Guanyin, Danshui, and Yilan, and 2) dust storm events with visibility less than 1 km for 24 hours in any of three neighboring First Global GARP Experiment-type ground stations [31]. After 2000, the Taiwan Environmental Protection Agency (TWEPA) became the official organization to define, monitor, and predict ADS. They utilize three distinct steps in order to categorize storm events in Taiwan as ADS and to predict their arrival time. First, the Weather Integration and Nowcasting System is consulted to confirm the occurrence of ADS in Mongolia and China. Second, the Moderate Resolution Imaging Spectroradiometer remote data and several models of ADS are used to track the transport of ADS and to predict the probability of the arrival of ADS in Taiwan. Third, if the data confirm that ADS may blow to Taiwan, the TWEPA will issue an early warning with the estimated arrival date [32]. This study highlights and analyzes 76 storm events that were thus categorized as ADS during the period of 1997–2007 (see Table 1). These ADS events span a total period of 172 dust storm days. Proportionally, these dust storm days account for 4.28% of the entire study period that lasted for a total time span of 4017 days. In addition to the ADS data, ambient pollutants concentrations and temperature have been regularly monitored at the TWEPA stations across Taiwan since 1994. The temperature measurements used in this analysis are the daily observations at the Jhongshan air quality monitoring station located in the most populated area of Taipei City (see Figure 1).

**Table 1:** Dust storm days in Taipei City, 1997–2007.

Year	Date	# of days
1997	1/1, 3/7–3/8, 3/30, 4/8, 4/21, 4/27–4/28,	8
1998	1/4, 2/13, 2/18–2/19, 3/7, 3/19, 3/30, 4/4, 4/15, 4/17–4/19, 4/24–4/26, 5/1, 11/5, 12/15	18
1999	1/27, 2/19, 3/8–3/9, 3/26, 4/7, 4/13, 11/25	8
2000	3/6–3/7, 3/24–3/25, 3/28–3/29, 4/6, 4/8, 4/10–4/11, 4/15–4/16, 4/22, 4/27–4/28, 5/1, 5/3–5/4, 5/13–5/18, 12/24	25
2001	1/13–1/15, 2/1, 2/16–2/17, 2/21–2/25, 3/1–3/7, 4/12–4/14, 5/1–5/2	23
2002	2/11–2/12, 3/6–3/9, 3/23–3/24, 3/31–4/1, 4/8–4/15, 4/17–4/19	21
2003	2/18–2/19, 2/23–2/25, 3/6–3/9, 3/25–3/30, 4/25–4/28	19
2004	1/1–1/4, 1/13–1/14, 1/21–1/22, 1/24–1/25, 2/6–2/12, 2/14–2/16, 2/26–2/27, 3/3–3/7, 4/2–4/4	30
2005	3/18–3/19, 11/29–11/30, 12/21–12/22	6
2006	3/19–3/20, 3/29–3/30, 4/20–4/21	6
2007	1/28–1/29, 4/2–4/3, 4/17–4/18, 12/30–12/31	8

Data source: These ADS dates were defined by two databases in the CCU (1997–1999) and TWEPA (2000–2007). The criteria of the determination for ADS dates can refer to the Dust Storm Data subsection.

### Study Area

Taipei City, located in northern Taiwan, is the country’s capital and largest metropolitan area with a population of more than six million inhabitants. Topographically, it is the second largest basin in Taiwan, bounded by the Yangming Mountains to the north, the Linkou mesa to the west, and Snow Mountains to the southeast. The basin region along the rivers is more populated than regions near the mountains, creating serious air pollution problems due to its heavy traffic. In the past decade, Taipei’s metropolitan rapid transit system (MRT) has effectively reduced cross-district traveling times within Taipei, and therefore, it has increased the accessibility to major medical facilities. Figure 1 displays the Taipei City map and essential urban information relevant to this study, including geographic topography, districts, major medical facilities, and the MRT. The clinic visit data are aggregated with respect to the 12 districts across Taipei City.

### Spatiotemporal Modeling

In this study, assuming 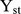 is the number of daily children’s respiratory clinic visits at calendar time 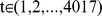 in district 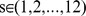, this outcome variable would follow a Poisson distribution by 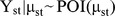 with the expected value 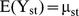 and variance 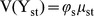, where 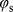 is an over-dispersion parameter representing the variation of clinic visits unable to be calculated by statistical models. A vector of dust storm lag index (DSLI) contains eight dummy variables to represent 0-lag to 7-lag days of ADS. Moreover, a vector of day-of-week (DOW) dummy variables from Monday to Saturday is used to control short-term temporal autoregressive correlations. The B-spline with a second order random walk prior is used in a time smoother 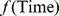 for calendar time to consider long-term autoregressive correlations and in a temperature smoother 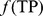 to control nonlinear weather confounding effect. Therefore, a model framework can be constructed by applying a Bayesian structural additive regression (STAR) modeling approach:


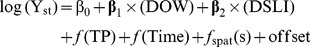
(1)

where 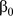 is an intercept for interpreting the overall association for all districts. The parameters 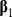 and 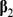 are a 1×6 vector with six coefficients of day-of-week variables and a 1×8 vector with eight coefficients of DSLI variables. The offset is the logarithm of the district-level population based on the 2000 Census.

In particular, a spatial function 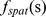 was appended to consider potential spatial autocorrelations among the 12 districts. It is actually a Markov random field [33] achieved by a conditional autoregressive prior to following a normal distribution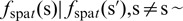 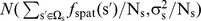, where 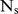 is the number of adjacent districts s’ connected to district s, and 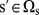 represents that the district s’ belongs to the set of the neighboring districts 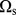 of district s. Note that the geographic information system in this study is boundary data, and that the definition of two connected districts means that they share parts of boundaries. In the spatial function, the unknown variance, 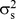, and smoothing parameter are assumed to follow an inverse Gamma distribution IG(0.001, 0.001). The spatial effect can be explained by the relative rate (RR) in district s compared with the mean value for the whole population controlling for spatial autocorrelations [34]. Maps of the spatial function at the district level were presented to visualize the geographic distribution of RR. Spatial effects were also classified into three groups according to their posterior probabilities with respect to the number 1 in the following manner: (i) 80% of the posterior distribution below 1 representing a significantly lower RR than the mean level for the Taipei City area; (ii) 80% of the posterior distribution above 1 representing a significantly higher RR than the mean level for the Taipei City area; (iii) the other districts representing a non-significant difference of RR compared to the mean level for the Taipei City area.

All unknown parameters in this STAR model were estimated by an empirical Bayesian approach using a restricted maximum likelihood (REML) estimation technique [35]. The REML method is a substitution of the maximum likelihood method that avoids the loss of degrees of freedom while estimating fixed effects, and prevents estimators toward zero with biases. Demographic analyses were generated using SAS v9.2 software [36], and the model-based estimation procedure was accomplished in the software package BayesX v2.01 [37]. A p-value <0.05 was considered statistically significant in estimated coefficients.

## Results

Table 2 depicts the regional distribution of children’s average daily respiratory clinic visits in Taipei City from 1997–2007. During ADS events (lag 0), the daily visit average reached its highest value with 1484.30 clinic visits (SD = 477.40) in the Shihlin District, while the Datong District only had 399.67 (SD = 161.72) clinic visits per day. The daily average of clinic visits increased during the 1-day lag at the end of ADS events, with the largest increment occurring in the Daan District (153.62 daily clinic visits). The Jhongjheng District only featured a 27.58 increase. After a 1-day lag, clinic visits decreased in most areas, however this falling trend was inconsistent. Some areas had a higher average during the 5-day lag or 7-day lag. Moreover, according to the daily records at the Jhongshan air quality monitoring station, the average concentration of PM_10_ was 90.64 µg/m^3^ during dust storm days, which was significantly higher than the average concentration during non-dust storm days (p-value <0.0001), as shown in Table 3. The average concentration of O_3_ during dust storm days was 5.61 ppb higher than that during non-dust storm days significantly (p-value <0.0001). This finding confirms that during ADS events as verified by the CCU and TWEPA, higher concentrations of two important air pollutants (i.e., PM_10_ and O_3_) were measured.

**Table 2:** Daily average children’s respiratory clinic visits within 12 Taipei City districts, 1997–2007.

District	Lag 0	Lag 1	Lag 2	Lag 3	Lag 4	Lag 5	Lag 6	Lag 7
Songshan	965.47	1021.01	1026.54	1032.12	1012.05	1003.30	1000.94	1022.76
	(265.40)†	(277.85)	(268.96)	(197.34)	(258.99)	(237.52)	(264.23)	(254.23)
Daan	1390.45	1544.07	1568.15	1544.26	1533.63	1539.41	1501.92	1482.48
	(500.60)	(566.66)	(556.80)	(469.63)	(576.84)	(511.05)	(526.60)	(524.48)
Datong	399.67	432.73	459.15	444.85	444.31	443.38	434.77	449.74
	(161.72)	(160.29)	(177.51)	(143.87)	(179.67)	(155.48)	(170.44)	(167.57)
Jhongshan	1283.78	1378.96	1424.59	1390.27	1380.77	1382.75	1350.28	1323.98
	(465.89)	(528.91)	(506.87)	(443.06)	(503.57)	(483.00)	(494.20)	(458.95)
Neihu	1278.21	1400.53	1428.16	1403.61	1397.21	1400.11	1373.70	1386.88
	(411.69)	(463.82)	(464.99)	(359.31)	(448.30)	(438.58)	(428.06)	(443.15)
Nangang	553.05	584.36	596.82	583.45	570.15	584.66	583.83	570.26
	(176.20)	(204.50)	(194.47)	(167.83)	(183.78)	(174.25)	(184.00)	(181.75)
Shihlin	1484.30	1604.77	1627.00	1589.94	1578.60	1583.27	1560.02	1550.04
	(477.40)	(548.67)	(520.92)	(434.24)	(522.40)	(485.19)	(499.14)	(499.69)
Beitou	1246.64	1355.39	1360.04	1357.71	1329.35	1327.86	1321.17	1280.04
	(427.35)	(457.99)	(446.99)	(374.80)	(451.62)	(405.07)	(435.30)	(411.12)
Sinyi	976.27	1064.72	1095.60	1067.12	1071.74	1059.79	1052.68	1060.38
	(302.20)	(337.22)	(332.92)	(260.57)	(329.34)	(303.71)	(295.59)	(307.20)
Jhongjheng	924.74	952.32	985.72	982.88	940.55	953.96	945.58	892.30
	(353.96)	(372.18)	(362.77)	(320.12)	(359.16)	(346.83)	(364.38)	(337.27)
Wanhua	866.25	959.19	973.10	941.47	953.13	947.93	932.32	927.58
	(296.49)	(343.05)	(325.99)	(271.42)	(329.86)	(303.55)	(321.07)	(317.90)
Wunshan	1233.33	1323.68	1340.56	1313.18	1307.82	1312.39	1282.19	1302.18
	(376.84)	(427.47)	(388.18)	(309.02)	(387.81)	(373.03)	(353.82)	(390.76)

† Standard deviation.

**Table 3:** Mean levels of air pollutants on dust storm days and non-dust storm days in Taipei City, 1997–2007.

Air pollutant	Dust storm days(n = 172)	Non-dust storm days(n = 3845)	P-value†
CO (ppm)	0.99±0.43	0.99±0.38	0.88
NO_x_ (ppb)	51.32±23.16	51.59±20.58	0.88
O_3_ (ppb)	24.17±7.67	18.56±8.19	<0.0001
PM_10_ (µg/m^3^)	90.64±42.18	52.74±23.53	<0.0001
SO_2_ (ppb)	4.46±2.67	4.28±2.32	0.39

† T-test.

Table 4 shows the increased percentage of RR of children’s respiratory clinic visits in certain DOW variables. Monday had the significantly highest increased percentage 37.55% (p-value <0.0001; 95% CI = 37.44, 37.65) compared to Sunday in preschool children and 37.88% (p-value <0.0001; 95% CI = 37.73, 38.08) in school children. Saturday was the second leading DOW variable, and the percentage increase of RR in school children (10.76%, 95% CI = 10.63, 10.88) was almost twice the percentage in preschool children (5.66%, 95% CI = 5.56, 5.75). Wednesday had the lowest percentage increase of RR of only 0.56% (p-value <0.0001; 95% CI = 0.47, 0.65) in preschool children, while the percentage largely inflated to 7.64% (p-value <0.0001; 95% CI = 7.51, 7.77) in school children. For all children, the greatest percentage increase of RR was 37.64%, occurred on Monday (p-value <0.0001; 95% CI = 37.55, 37.72).

**Table 4:** Percentage change in rates of daily children’s respiratory clinic visits in Taipei City, 1997–2007 [% (95% CI)].

Variable		Preschool children		School children		All children	
DOW	Monday	37.55	(37.44, 37.65)	37.88	(37.73, 38.08)	37.64	(37.55, 37.72)
	Tuesday	0.81	(0.72, 0.90)	−1.11	(−1.23, −0.99)	0.11	(0.04, 0.18)
	Wednesday	0.56	(0.47, 0.65)	7.64	(7.51, 7.77)	2.90	(2.83, 2.98)
	Thursday	1.35	(1.26, 1.44)	−2.09	(−2.21, −1.97)	0.18	(0.10, 0.25)
	Friday	3.35	(3.26, 3.44)	2.33	(2.20, 2.45)	2.98	(2.91, 3.05)
	Saturday	5.65	(5.56, 5.75)	10.76	(10.63, 10.89)	7.39	(7.32, 7.47)
	Sunday	Reference level		Reference level		Reference level	
DSLI	Lag 0	−2.53	(−2.69, −2.36)	−6.28	(−6.50, −6.06)	−3.66	(−3.79, −3.53)
	Lag 1	−2.12	(−2.34, −1.89)	−1.66	(−1.97, −1.34)	−2.05	(−2.23, −1.87)
	Lag 2	2.12	(1.88, 2.35)	0.73	(0.39, 1.06)	1.78	(1.59, 1.98)
	Lag 3	2.19	(1.95, 2.43)	3.17	(2.83, 3.52)	2.40	(2.20, 2.59)
	Lag 4	0.63	(0.39, 0.88)	0.72	(0.37, 1.07)	0.66	(0.45, 0.86)
	Lag 5	1.01	(0.75, 1.26)	2.44	(2.07, 2.81)	1.74	(1.53, 1.96)
	Lag 6	−1.07	(−1.33, −0.81)	−0.84	(−1.21, −0.47)	−1.01	(−1.23, −0.80)
	Lag 7	2.18	(1.90, 2.46)	3.20	(2.81, 3.60)	2.26	(2.03, 2.49)
	The other days	Reference level		Reference level		Reference level	

Abbreviation: DOW  =  day-of-week; DSLI  =  dust storm lag index.

The association between ADS and children’s respiratory clinic visits was not positive until the second day after ADS, suggesting the percentage increase of RR for preschool children was −2.53% (p-value <0.0001; 95% CI = −2.69, −2.36), while it was much lower in school children by −6.28% (p-value <0.0001; 95% CI = −6.50, −6.06). The negative association lasted through the 1-day lag, and became positive from the 2-day lag to the 7-day lag, except for the 6-day lag. Among lag days with positive associations, preschool children had the highest 2.19% (p-value <0.0001; 95% CI = 1.95, 2.43) at the 3-day lag; meanwhile for school children, RR at the 7-day lag reached its highest percentage increase by 3.20% (p-value <0.0001, 95% CI = 2.81, 3.60). Regardless of age stratification, the strongest association happened at the 3-day lag with a 2.40% (p-value <0.0001; 95% CI = 2.20, 2.59) increase in RR for all children.

Figure 1 depicts the distribution of spatial effects attributed to children’s respiratory clinic visits in Taipei City. In most districts with preschool children or school children, positive risk of increased clinic visits was prevalent. The range of spatial effect in preschool children was (−0.37, 0.27), and it was wider than that for school children (−0.22, 0.13). Out of all the 12 districts studied, the Jhongshan District displayed the strongest spatial effect contributed to respiratory clinic visits for both preschool and school children. The positive spatial effect in preschool children’s clinic visits was uniformly distributed in the following districts: the Beitu, Shihlin, Neihu, Nangang, and Wanshan District; however, no specific pattern described the spatial heterogeneity in school children’s clinic visits. The maps of 80% posterior probability show that 6 of 12 districts demonstrated a significantly positive spatial effect in both preschool children and school children. Combing two groups, 7 of 12 districts had a significantly positive spatial effect, which locations are identical to the finding in preschool children.

## Discussion

Due to high PM concentrations and unusual PM compositions during ADS, ADS and their occurrences have been considered to pose a high risk to human health. Recent studies have demonstrated potential health risks associated with ADS in terms of higher mortality rates and hospital admissions [21], [38]. Some studies have evaluated the biological plausibility of the ADS to induce adverse health effects. These studies have shown that the particles in ADS can exert toxicological effects on the respiratory system, such as causing pulmonary inflammation and inducing cytotoxicity in rat alveolar cells [39]–[44]. However, other epidemiological studies have noted that the relationship between ADS and adverse health effects, particularly respiratory diseases, is at best uncertain or statistically insignificant [3], [21], [45]–[48]. One plausible explanation of these inconsistencies may be that the health assessment measures used to evaluate the health impact of the ADS did not adequately capture the health effects. For instance, some of the health measures utilized only accounted for severe cases that required inpatient care, and consequentially, negative environmental events such as ADS may not necessarily induce such severe medical conditions. Furthermore, these previous analyses were based upon observations from limited hospitals [21], [25], [45], [48], and reflected severe health conditions that were exhibited by the most vulnerable individuals within a general population. In contrast, this study provides complete ambulatory and emergency service utilization information from the NHI, and hopefully, better captures the potential health impact of ADS events on the general population.

In recent decades, the importance of spatiotemporal analysis has been emphasized in environmental epidemiological research, especially in quantifying uncertainties in space-time health and exposure data as well as capturing the resulting impact on the estimates of these associations [28], [49], [50]. Although temporal health impacts caused by ADS have been extensively investigated, previous studies have seldom considered the spatial heterogeneity of such health data in which the geographic disparity may be derived from geographical variations of medical resources and exposure levels.

In response, this study implemented the STAR modeling approach for the spatiotemporal analysis of children’s clinic visits related to ADS. The model identifies temporal patterns of a time process by accounting for linear and nonlinear explanatory variables similar to many time series models, such as the generalized additive model [51]. Moreover, the STAR model reveals the spatial heterogeneity independent of temporal variations by using Markov random fields. In addition, the Bayesian framework of the STAR model allows feasibility to account for the parameter’s uncertainty. This novel approach provides a more comprehensive perspective on the impact of ADS on children’s clinic visits for respiratory illnesses.

Previous studies have demonstrated different results in the temporal lag effects of adverse human health related ADS. For instance, hospital admissions were prominent 2 days after ADS in one asthma study [45]. Also, in another study, a positive influence was noted between ADS and ischemic stroke hospital admissions on the third day following a dust storm event [19]. At the 1-day lag, the relative risk of the association between ADS and cardiovascular disease hospital admissions is also increased [14]. However, these associations were statistically insignificant. In contrast, hospital admissions records in Taipei City noted a significant increase in ischaemic heart disease admissions at the 2-day lag and asthma admissions at the 3-day lag [21]. Meanwhile, total respiratory diseases at the 3-day lag and upper respiratory tract infection in males at the 4-day lag were significant in Minqin City, China [48]. As noted, these findings were mostly based upon the analysis of more severe health measures. This study conducted a population-based study and found that children’s respiratory health can be affected by ADS. This impact significantly occurred during most days within a week after a dust storm event. The elevated rates for children’s respiratory clinic visits after a dust storm began at the 2-day lag and attained its highest impact at the 3-day lag for preschool children and the 7-day lag for school children.

Several of the following considerations may provide plausible explanations for such an increased rate of children’s respiratory clinic visits: First, the impact of the ADS may not necessarily incite immediate respiratory illness or illness severe enough to necessitate the patient to seek medical services. A latency period may exist between the adverse environmental influence and the onset of respiratory symptoms requiring the need for medical services. Second, the adverse weather conditions that exist during ADS, such as strong winds and low visibility [52], often prevent citizens from going out. Third, the increasing popularity of ADS forecasting by the media and governmental agency may increase the population awareness of ADS and their potential health effects. Fourth, since over-the-counter pharmaceuticals are easily accessible and inexpensive in Taiwan, many Taiwanese residents may prefer initiating treatment of their symptoms and their children’s symptoms with these products before seeking medical treatment at a clinic, especially if they perceive that their condition is not serious. However, if unsuccessful, treatment with these over-the-counter medications could also account for the lag time noticed in the children’s respiratory clinic visits following ADS. Table 4 notes that the consecutive elevated risks may only apply to the children because of their vulnerability to ambient pollutants. Further studies should assess the ADS health impact on other age groups. Table 4 also shows that school children were affected by ADS much easier than preschool children. This may be explained by the fact that Taiwanese schools were not suspended during ADS, and school children may have experienced higher exposures and elevated concentrations of heavy metals and ambient PM compositions than their preschool counterparts who more likely stayed at home [4], [53], [54]. This type of exposure has been closely associated with the reduction of pulmonary functions in children [23]. Further studies should investigate the temporal fluctuation of daily relative risks that may result from the cross-infection of respiratory diseases among children or the influence payment policy of the Taiwanese NHI system.

Interestingly, the day-of-week has been considered as a meaningful confounding factor for clinic visits in Taiwan. It is important to know that local ambulatory service is commonly rendered on a “first-come, first-serve” basis, and that physician appointments are not necessary for the regular weekday schedule. Access to any level of healthcare facility or provider is therefore unconstrained during the week. However, weekend medical services must be justified by severe symptoms and result in higher co-payment requirements (out-of-pocket amount) from the NHI. Thus, the service schedule and payment system are important factors affecting the timing of medical-care-seeking behavior. Thus, the temporal pattern of clinic visits is closely associated with this weekend effect.

In Taiwan, the majority of the medical services in hospitals and clinics are closed from Saturday afternoon until Monday morning. Therefore, there is a strong incentive to visit clinics on Fridays and Saturday (before weekend effect), and on Monday (after weekend effect). The day-of-week clinic visit pattern observed in this study is quite consistent with medical care-seeking pattern that has resulted under the current national health care delivery system. Moreover, the highly elevated RR on Monday essentially comprised those patients seeking medical treatment from Saturday to Monday, especially in the case of children who are incapable of accessing clinic care independently and are dependent on a parent’s working schedule which allows for only nighttime availability [55].

**Figure 2:**
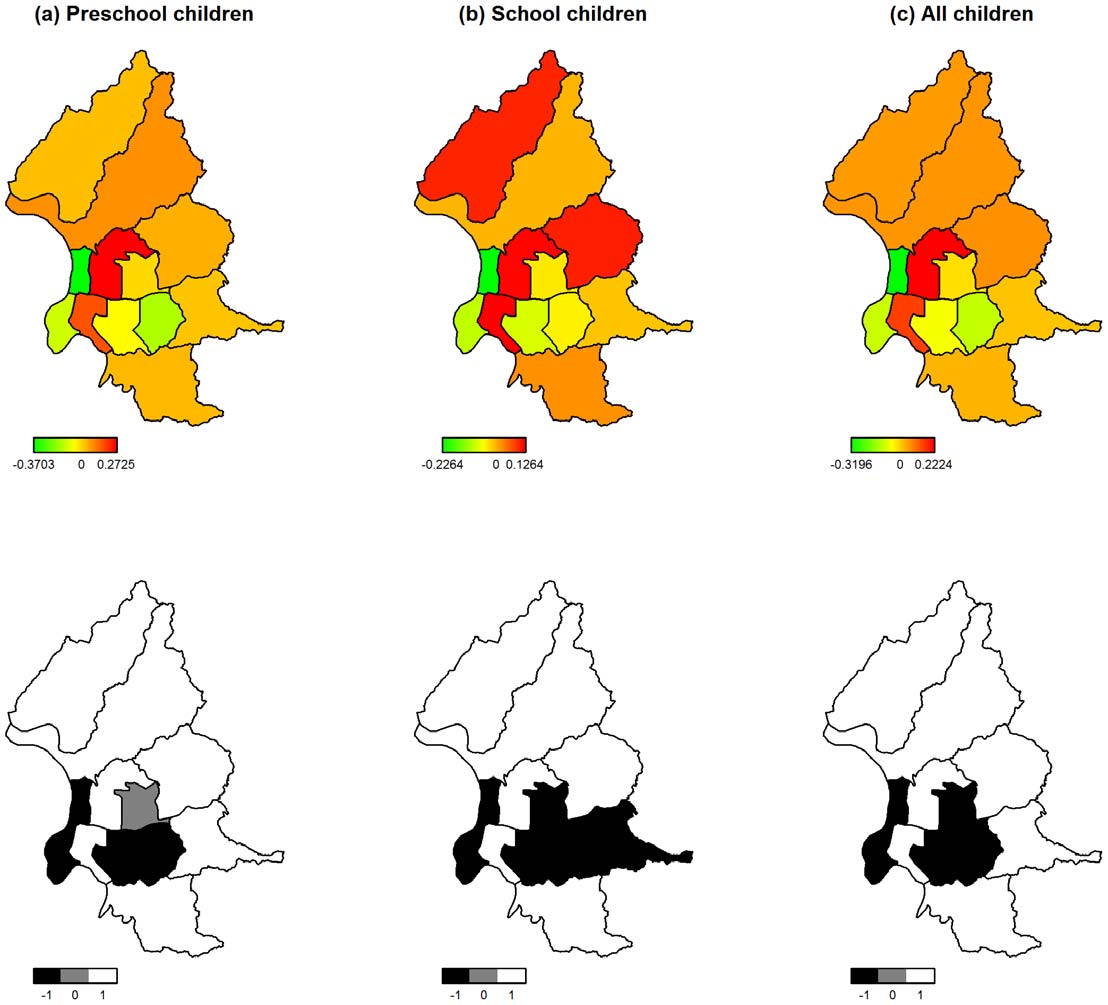
Spatial effects with 80% posterior probability for (a) preschool children, (b) school children, and (c) all children. Districts shaded by white color showed significantly positive spatial effect, whereas districts shaded by black color depicted significantly negative spatial effect. Grey color represented non-significant spatial effect in the district.

The spatial heterogeneity of clinical visits also reflects children’s respiratory clinic visits (see Figure 2). Compared to Figure 1, the districts with elevated rates were closely linked to the areas with multiple urban medical centers, especially those along the MRT lines, implying that a high usage of ambulatory and emergency services for children’s health might logically occur in these districts. Because the NHI program is characterized by its low co-payments and open access to providers without choice restrictions, it encourages those insured under the current Taiwan NHI system to seek care in these medical centers with minimal personal financial impact. Consequently, each person in Taiwan averages 14.2 clinic visits per year. In addition, some people may seek treatment of common diseases in hospitals or even tertiary medical centers, rather than clinics [24]. Further study is required to investigate the relationship between the identified spatial heterogeneity and the locations of medical centers in the study area.

### Conclusion

In summary, the spatiotemporal analysis presented in this study identifies the temporal pattern of the health risks during and after ADS and analyzes them day-by-day by considering the spatial confounding factor. The study results clearly show significant and increased rates for respiratory clinic visits in the studied population of children over time in 5 of 7 days after ADS. The findings of this population-based study can provide governmental agencies with an important reference source in order to plan and implement policies that can help to both protect children from the possible adverse health effects of ADS and to provide care related to such health effects.
